# Loss of LCN2 Function Ameliorates Glucocorticoid‐Induced Muscle Atrophy via Remodeling the Extracellular Matrix

**DOI:** 10.1002/advs.77189

**Published:** 2026-08-18

**Authors:** Hongwei Shi, Xiaojing Hao, Yi Yan, Fengyang Li, Yaru Zhang, Zixin Li, Wenjuan Xie, Jiayin Lu, Xiaomao Luo, Yanjun Dong, Haidong Wang, Juan Wang

**Affiliations:** ^1^ College of Veterinary Medicine Shanxi Agricultural University Jinzhong Shanxi People's Republic of China; ^2^ College of Veterinary Medicine China Agricultural University Beijing People's Republic of China; ^3^ Department of Nephrology Shanghai General Hospital Shanghai Jiaotong University School of Medicine Shanghai People's Republic of China

**Keywords:** extracellular matrix, glucocorticoid receptor, Lipocalin 2, MMP9, muscle atrophy

## Abstract

Chronic glucocorticoid (GC) exposure is the leading clinical cause of skeletal muscle atrophy, steroid myopathy, and secondary sarcopenia, triggering irreversible motor function decline, disease progression, and elevated all‐cause mortality. The underlying pathological mechanisms remain unclear, and no safe and effective targeted interventions are currently available. This study identifies Lipocalin 2 (LCN2) as a pivotal driver of GC‐induced muscle atrophy. Using multi‐omics analysis, dexamethasone‐induced mouse models, primary myotube models, and gain/loss‐of‐function assays, we found that LCN2 was the most strikingly upregulated factor in atrophic muscle, and that its transcription was directly activated by glucocorticoid receptor (GR) binding to the conserved glucocorticoid response element (GRE) in its promoter. Muscle‐specific LCN2 overexpression disrupts extracellular matrix (ECM) homeostasis and triggers severe muscle atrophy and motor dysfunction, whereas LCN2 silencing markedly alleviates GC‐induced ECM injury and atrophy without impairing normal muscle homeostasis. Mechanistically, the interaction of LCN2 with matrix metalloproteinase 9 (MMP9) triggers ECM dysregulation and consequent focal adhesion kinase (FAK) signaling inactivation, which represses the PI3K‐Akt‐mTOR anabolic cascade and activates FoxO‐driven catabolic signaling, thereby leading to dysregulated muscle protein metabolism. This study reveals the core pathogenic role of the LCN2‐MMP9‐ECM‐FAK axis in GC‐induced muscle atrophy, providing a promising novel therapeutic target for steroid myopathy.

## Graphical abstract

Glucocorticoids transcriptionally activate LCN2 expression via GR nuclear translocation. Secreted LCN2 binds MMP9 to degrade skeletal muscle ECM collagen, blocks integrin‐mediated mechanotransduction, bidirectionally disrupts muscle protein homeostasis, and reveals a novel target for steroid‐induced muscle atrophy.

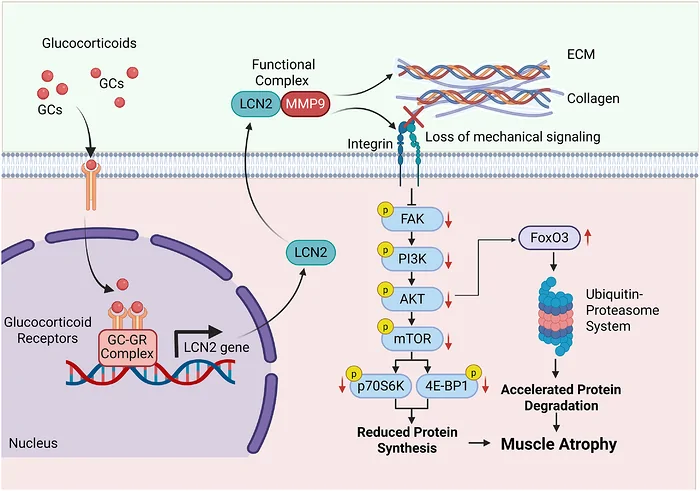

## 1 Introduction

Skeletal muscle atrophy is a common and severe complication caused by aging, chronic diseases, disuse, and long‐term glucocorticoid (GC) exposure, and is characterized by muscle mass loss, impaired contractile function, and decreased motor performance [1, 2, 3, 4]. As essential anti‐inflammatory and immunosuppressive agents, GCs are widely used for autoimmune diseases, allergic disorders, and organ transplantation; however, their clinical utility is severely limited by iatrogenic muscle wasting [5]. Critical illnesses such as sepsis and cachexia also sharply elevate endogenous GC levels, further inducing atrophy [6].

In skeletal muscle, GCs bind to the glucocorticoid receptor (GR), promoting its conformational change and nuclear translocation, and subsequently target glucocorticoid response elements (GREs) within the promoters of downstream genes [7]. This GR–GRE interaction initiates extensive transcriptional reprogramming and upregulates key atrophy‐related transcription factors, among which the FOXO family serves as the central regulator. Activated FOXOs directly drive the transcription of atrophic genes and strongly induce the muscle‐specific E3 ubiquitin ligases Atrogin‐1 and MuRF1, which degrade myofibrillar proteins via the ubiquitin–proteasome system (UPS), thereby accelerating protein catabolism [8, 9]. Simultaneously, GCs suppress anabolic signaling through the AKT/mTOR pathway, further inhibiting protein synthesis [10]. Despite these mechanistic insights, effective therapeutic targets for GC‐induced muscle atrophy remain poorly defined.

The extracellular matrix (ECM) is a dynamic three‐dimensional network of collagens, proteoglycans, and adhesive glycoproteins that provides mechanical support and regulates anabolism and mechanotransduction through integrin‐mediated signaling, thus preserving skeletal muscle homeostasis [11, 12]. Pathological disruption of ECM balance activates pro‐atrophic cascades and contributes to muscle damage [13]. Matrix metalloproteinases (MMPs) are essential for ECM degradation; their overactivation during injury and disuse excessively degrades ECM components and compromises myofiber structure and function [14, 15]. Although ECM dyshomeostasis is a critical driver of muscle atrophy, whether GCs trigger downstream atrophic signals by inducing ECM degradation and structural disorganization remains largely unknown.

Lipocalin 2 (LCN2) is a multifunctional secreted glycoprotein involved in iron chelation, innate immunity, and tissue remodeling and broadly participates in skeletal muscle development, injury repair, regeneration, and metabolic disease pathophysiology [16, 17, 18, 19]. We previously demonstrated that LCN2 is markedly upregulated after muscle injury and drives mitochondrial dysfunction by upregulating ACOD1, leading to reactive oxygen species accumulation, mitochondrial membrane potential collapse, ferroptosis, and ultimately impaired myogenesis [20]. However, its role in the GC‐induced muscle atrophy remains unknown. Here, we identify LCN2 as a direct transcriptional target of the GR and show that GC stimulation directly activates LCN2 transcription in muscle. Using AAV9‐mediated muscle‐specific LCN2 overexpression and knockdown in mice, we found that LCN2 overexpression disrupts ECM homeostasis and induces pronounced muscle atrophy, whereas LCN2 silencing restores ECM homeostasis by suppressing MMP9‐mediated excessive ECM degradation and effectively ameliorates GC‐induced muscle atrophy. These findings provide novel insights into the pathogenesis of GC‐induced muscle atrophy.

## 2 Materials and Methods

### 2.1 Animal Models

All animal procedures were conducted in strict compliance with the guidelines approved by the Institutional Animal Care and Use Committee of Shanxi Agricultural University (Approval No. SXAU‐EAW‐2025M.ZS.006010443). Male C57BL/6J mice were maintained in a specific pathogen‐free (SPF) animal facility at our institution with ad libitum access to standard rodent chow and water under a 12‐h light/dark cycle.

#### 2.1.1 Establishment of a Skeletal Muscle‐Specific LCN2 Overexpression Mouse Model

The full‐length mouse *Lcn2* coding sequence was synthesized and subcloned into the AAV9:CMV‐MSC‐3×Flag‐EF1‐ZsGreen‐T2A‐Puro vector by Hanheng Biotechnology (Shanghai, China). Eight‐week‐old male C57BL/6J mice were anesthetized and injected intramuscularly with empty vector or *Lcn2*‐carrying AAV9 vector at 1 × 10^1^ ^2^ vg per mouse. Four weeks later, the mice were euthanized, and the gastrocnemius (GAS), tibialis anterior (TA), and quadriceps (QU) muscles were collected for analysis.

#### 2.1.2 Establishment of a Muscle‐Specific LCN2 Knockdown Mouse Model

For GC‐induced muscle atrophy models and AAV9‐mediated muscle‐specific LCN2 knockdown, 8‐week‐old male C57BL/6J mice were anesthetized and intramuscularly injected with an empty vector or HBAAV2/9‐MHCK7‐Lcn2 shRNA (synthesized by Hanheng Biotechnology, Shanghai, China) at a dose of 1 × 10^12^ vg per mouse.

#### 2.1.3 Establishment of a Mouse Muscle Atrophy Model and Administration of Recombinant MMP9 Protein

The mice received daily intraperitoneal injections of dexamethasone (HY‐14648, MCE, China; molecular weight: 392.46 g/mol; purity: 99.86%) at 25 mg/kg for 10 consecutive days to induce muscle atrophy. Alternatively, dexamethasone (1 mg/kg/day) was administered via intragastric gavage for 1 consecutive month to establish a prolonged atrophy phenotype. For the rescue experiment, recombinant activated mouse MMP9 protein (909‐MM, R&D Systems, USA) was co‐administered intraperitoneally at 5 µg/mouse/day throughout the Dex treatment course. The mice were sacrificed 24 h after the last injection, and skeletal muscle samples were collected for further experiments.

#### 2.1.4 Skeletal Muscle Function Assessment

##### 2.1.4.1 Limb Grip Strength

Grip strength was measured using a muscle force tester (SANS Biotechnology, Nanjing, China). The mice were placed on a horizontal grid and gently pulled backward for 10 trials, and the peak force before release was recorded in Newtons (N).

##### 2.1.4.2 Inverted Grid Hanging Test

The inverted grid test was used to evaluate skeletal muscle strength and endurance. Mice were placed in the center of an inverted metal grid with a soft cushion placed 30 cm below the grid. The latency to fall was recorded for statistical analysis.

##### 2.1.4.3 Treadmill Exhaustive Running Test

Exercise endurance capacity was evaluated using a motorized treadmill system (SANS Biotechnology, Nanjing, China). The mice underwent 2–3 days of acclimatization training on a treadmill prior to formal testing. During the test, the mice ran on a treadmill at 13% incline, with the speed incrementally increased by 5 cm/s every 2 min until exhaustion. Exhaustion was operationally defined as the inability to resume running despite gentle prodding and remaining on the electrical stimulus grid for > 10 consecutive seconds. Time to exhaustion and total running distance were recorded for each mouse.

### 2.2 Cell Culture

C2C12 cells (CRL‐1772; ATCC, USA) and human primary skeletal muscle myoblasts (PRI‐H‐00152, ZQXZbio, China) were maintained at 37°C in a humidified atmosphere containing 5% CO_2_. C2C12 cells were cultured in high‐glucose DMEM (11965092, Gibco, USA) supplemented with 10% fetal bovine serum (10099141, Gibco, USA). Human primary skeletal muscle myoblasts were grown in dedicated primary skeletal muscle myoblast complete medium (PCM‐H‐177, ZQXZbio, China) according to the manufacturer's protocol. Both cell types were switched to a differentiation medium containing 2% horse serum (S9050, Solarbio, China) for 4 days to form mature myotubes. To establish an in vitro skeletal muscle atrophy model, mature myotubes were treated with 50 µm dexamethasone for 24 h.

Lipofectamine 2000 (11668019, Invitrogen, USA) was used for plasmid and siRNA transfection according to the manufacturer's instructions. For LCN2 overexpression, fully differentiated myotubes were transfected with either the *Lcn2* overexpression plasmid construct or the corresponding empty vector control. For LCN2 knockdown, myotubes were transfected with *Lcn2*‐targeting siRNA (si‐*Lcn2*) or non‐targeting negative control siRNA. The transfection complexes were removed after 6 h of incubation, and the cells were replenished with fresh differentiation medium for subsequent experiments.

### 2.3 Co‐Immunoprecipitation

Four‐day‐differentiated C2C12 myotubes were transfected with *Lcn2* overexpression plasmid or empty vector. After 48 h, total protein was extracted using cold IP lysis buffer containing protease inhibitors. Equal amounts of protein were incubated with anti‐LCN2 antibody (AF1857, R&D Systems, USA) or control IgG (30000‐0‐AP, Proteintech, China) overnight at 4°C, followed by incubation with Protein A/G agarose beads (sc‐2003, Santa Cruz, USA) for 4 h. Immunocomplexes were eluted by boiling in SDS‐loading buffer after washing, and LCN2‐MMP8/MMP9 interactions were detected by western blotting.

### 2.4 Western Blotting

Protein levels in the muscle tissues and myotubes were detected using western blotting. Samples were lysed in ice‐cold RIPA buffer with protease and phosphatase inhibitors, and protein concentration was determined by BCA assay. Thirty micrograms of protein per sample were separated by SDS‐PAGE and transferred to PVDF membranes. Membranes were blocked with 5% non‐fat milk, incubated with primary antibodies overnight at 4°C, followed by incubation with HRP‐conjugated secondary antibodies. Protein bands were visualized with ECL substrate and quantified using ImageJ software with GAPDH as an internal reference. Detailed antibody information is provided in Table S1.

### 2.5 ChIP Assay

ChIP assays were conducted to detect GR binding to the *Lcn2* promoter in both Dex‐treated (+Dex) and untreated (−Dex) cells. Cells were cross‐linked with 1% formaldehyde for 10 min, and glycine was added to quench cross‐linking. Chromatin was sonicated into 200–500‐bp fragments, and lysates were incubated with anti‐GR antibody (86748SF, Cell Signaling Technology, USA) or control IgG (30000‐0‐AP, Proteintech, China) overnight at 4°C, followed by incubation with Protein A/G beads (9006S, Cell Signaling Technology, USA) for 4 h. After washing, chromatin was de‐crosslinked, and DNA was purified. Quantitative polymerase chain reaction (qPCR) with *Lcn2* promoter‐specific primers was performed to assess binding enrichment. Primer sequences are listed in Table S2.

### 2.6 Immunofluorescence Staining

Immunofluorescence staining was performed on C2C12 myotubes and mouse skeletal muscle tissues. Cells were fixed with 4% paraformaldehyde (G1101, Servicebio, China), permeabilized with 0.2% Triton X‐100 (ST1723, Beyotime, China), and blocked with 5% BSA. Samples were incubated with primary antibodies overnight at 4°C, followed by incubation with Alexa Fluor 488/594 (Invitrogen) secondary antibodies for 1 h at room temperature in the dark.

Muscle tissues were fixed in 4% paraformaldehyde, embedded in paraffin, and cut into 5‐µm sections. After dewaxing and antigen retrieval, sections were processed as described above, and nuclei were counterstained with DAPI (P0131, Beyotime, China). Images were captured by laser confocal microscopy, and fluorescence intensity and myofiber cross‐sectional area were quantified using ImageJ software.

### 2.7 Transcriptome Sequencing and Quantitative Real‐Time PCR

Total RNA was extracted from C2C12 myotubes and skeletal muscle tissues using TRIzol reagent. Qualified RNA samples were sent to BGI Genomics for transcriptome sequencing on the DNBSEQ platform. Raw reads were filtered to obtain clean reads and mapped to the mouse reference genome for quantification of gene expression. Differentially expressed genes (DEGs) were screened with |log_2_FC| ≥ 1 and FDR < 0.05, followed by Gene Ontology (GO) and Kyoto Encyclopedia of Genes and Genomes (KEGG) enrichment analysis.

For real‐time reverse transcription polymerase chain reaction (qRT‐PCR), 1 µg total RNA was reverse transcribed into cDNA. Relative gene expression was quantified via the SYBR Green method with β‐actin as the internal reference and calculated using the 2^−ΔΔCt^ method. All primer sequences are listed in Table S2.

### 2.8 In Vitro SUnSET Assay for Protein Synthesis

Newly synthesized proteins were labeled by incubating cells with puromycin (A605186, AmBeed, USA; molecular weight: 471.51 g/mol; purity: ≥99%) at a final concentration of 1 µg/mL for 30 min after treatment. Total protein was extracted, and the protein synthesis rate was determined by western blotting using an anti‐puromycin antibody, with GAPDH used as the internal normalization control.

### 2.9 Statistical Analysis

All experiments were independently repeated three times. Data are presented as means ± SEMs. Statistical analyses were performed using GraphPad Prism, version 8. The Student's *t*‐test was used for two‐group comparisons, and one‐way ANOVA with Tukey's post‐hoc test was used for multiple groups. Statistical significance was set at *P* < 0.05.

## 3 Result

### 3.1 LCN2 is Upregulated in GC‐Induced Mouse Muscle Atrophy

Considering the potential regulatory role of LCN2 in GC‐induced muscle atrophy, we systematically characterized its expression profile in the skeletal muscles of mice with GC‐triggered atrophy. Bioinformatics analysis of public datasets PXD027464 and GSE159952 revealed that *Lcn2* was the most prominently upregulated molecule at both mRNA and protein levels in the GAS of mice with dexamethasone (Dex)‐induced muscle atrophy, with a 163‐fold mRNA increase and 27.64‐fold protein upregulation (Figure 1A). We further validated this finding in human skeletal muscle using the public transcriptomic dataset GSE17992, which includes biopsies from healthy male volunteers treated with Dex for 4 days, and observed consistent upregulation of LCN2 mRNA (Figure 1A). To validate these findings, we established a high‐dose Dex‐induced mouse model of muscle atrophy. Hematoxylin and eosin (H&E) staining showed significantly reduced myofiber cross‐sectional area (CSA) in the skeletal muscle of Dex‐treated mice (Figure 1B), accompanied by markedly upregulated atrophy marker proteins Atrogin‐1 and MuRF1, and significantly downregulated myosin heavy chain (MyHC), a key myogenic protein (Figure 1C), confirming successful model establishment. We found that the LCN2 protein was upregulated in the TA, GAS, and QU of Dex‐induced mice (Figure 1D). In vitro, 50 µm Dex treatment significantly upregulated *Lcn2* mRNA and protein levels in cultured C2C12 myotubes (Figure 1E), with a notable increase in secreted LCN2 in the myotube culture supernatant (Figure 1F). To further verify the evolutionary conservation and clinical relevance of Dex‐induced LCN2 upregulation in skeletal muscle, we performed parallel validation experiments using differentiated human primary myotubes. Dex treatment dramatically increased the LCN2 mRNA levels in human primary myotubes, mirroring the robust transcriptional induction observed in murine C2C12 myotubes (Figure 1G). At the protein level, Dex challenge significantly upregulated intracellular LCN2 expression, along with a concurrent elevation of two key atrophy‐related E3 ubiquitin ligases, MuRF1 and Atrogin‐1 (Figure 1H), confirming that LCN2 upregulation is tightly coupled with a Dex‐triggered atrophic response in human muscle cells. Consistent with the findings in murine myotubes, the concentration of secreted LCN2 in the supernatant of human primary myotube cultures markedly increased following Dex administration (Figure 1I). Collectively, bioinformatic analysis combined with in vitro and in vivo results consistently confirm that Dex significantly induced LCN2 expression and secretion in skeletal muscle tissues and myotubes, suggesting that aberrant LCN2 upregulation is closely associated with the pathological progression of GC‐induced muscle atrophy.

**FIGURE 1:**
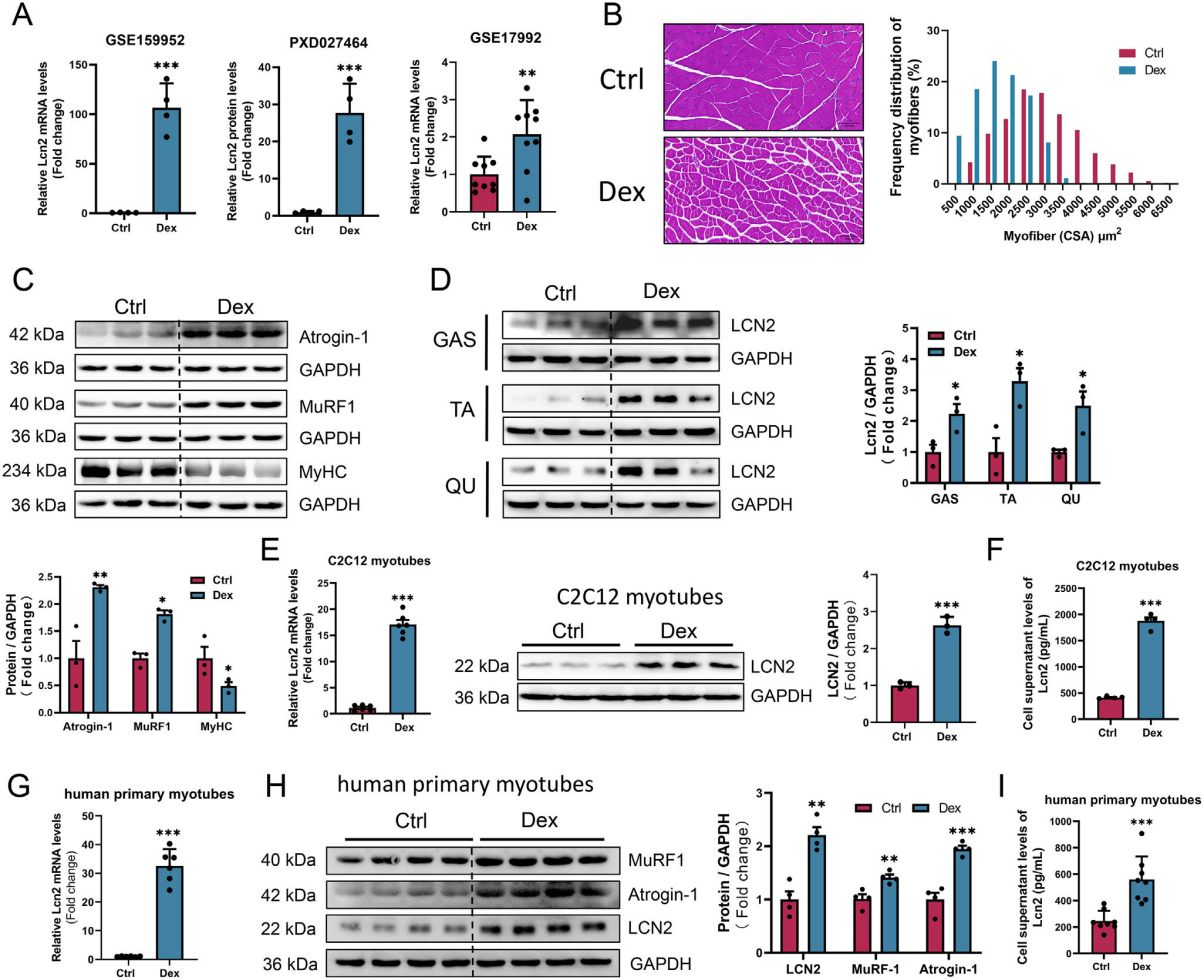
Glucocorticoid‐induced muscle atrophy is associated with elevated LCN2 levels. (A) Bioinformatics analysis of public datasets showing Lcn2 upregulation in Dex‐induced muscle atrophy: murine mRNA (GSE159952) and protein (PXD027464) in gastrocnemius, and human mRNA (GSE17992) in skeletal muscle biopsies. (B) Hematoxylin and Eosin (H&E) staining of gastrocnemius muscle sections from the control (Ctrl) group and Dex‐treated group, with the frequency distribution of myofiber cross‐sectional area (CSA) (Scale bar, 100 µm). (C) Western blot analysis of the muscle atrophy marker proteins Atrogin‐1, MuRF‐1 and myofiber maturation marker MyHC in the gastrocnemius muscle of mice from each group, with corresponding quantitative analysis of protein levels. GAPDH was used as the loading control (*n* = 3 per group). (D) Western blot analysis of LCN2 protein expression in the GAS, tibialis anterior (TA), and quadriceps (QU) muscles of mice from the control and Dex groups, with corresponding quantitative analysis. GAPDH was used as the loading control (*n* = 3 per group). (E) Quantitative real‐time PCR and Western blot analysis of Lcn2 mRNA (*n* = 6 per group) and protein (*n* = 3 per group) levels in differentiated C2C12 myotubes from the control and Dex groups. β‐actin and GAPDH were used as the reference gene and loading control, respectively. (F) ELISA detection of the concentration of secreted LCN2 in the culture supernatant of C2C12 myotubes from control and Dex‐treated groups (*n* = 4 per group). (G) Relative *LCN2* mRNA levels in human primary myotubes treated with or without Dex, as determined by quantitative real‐time PCR. β‐actin was used as the reference gene (*n* = 6 per group). (H) Western blot analysis of muscle atrophy markers (MuRF1, Atrogin‐1) and LCN2 protein in human primary myotubes from control and Dex groups. GAPDH was used as a loading control. (I) Concentrations of secreted LCN2 protein in the culture supernatants of differentiated human primary myotubes from the control (Ctrl) and Dex groups as detected by ELISA (*n* = 8 per group). All quantitative data are presented as the mean ± standard error of the mean (SEM). ^*^ *p*<0.05, ^**^ *p*<0.01, ^***^ *p*<0.001.

### 3.2 Exogenous LCN2 Induces Extracellular Matrix Damage and Skeletal Muscle Atrophy in Mice

To clarify the regulatory role of LCN2 in GC‐induced skeletal muscle atrophy, we generated a skeletal muscle‐specific LCN2 overexpression mouse model via intramuscular AAV9 injection. Compared with vector controls, AAV‐LCN2 mice exhibited overt atrophy of multiple skeletal muscle groups (GAS, TA, and QU) with significantly reduced tissue wet weight (Figure 2A). LCN2 overexpression also markedly impaired muscle function, as evidenced by decreased grip strength, grid hanging time, and treadmill running distance (Figure 2B). H&E staining revealed a significant reduction in myofiber CSA in AAV‐LCN2 mice (Figure 2C), which was consistent with in vitro data showing that LCN2 overexpression significantly decreased the diameter of C2C12 myotubes (Figure 2D). These in vivo and in vitro results collectively confirm that LCN2 directly induces myofiber atrophy.

**FIGURE 2:**
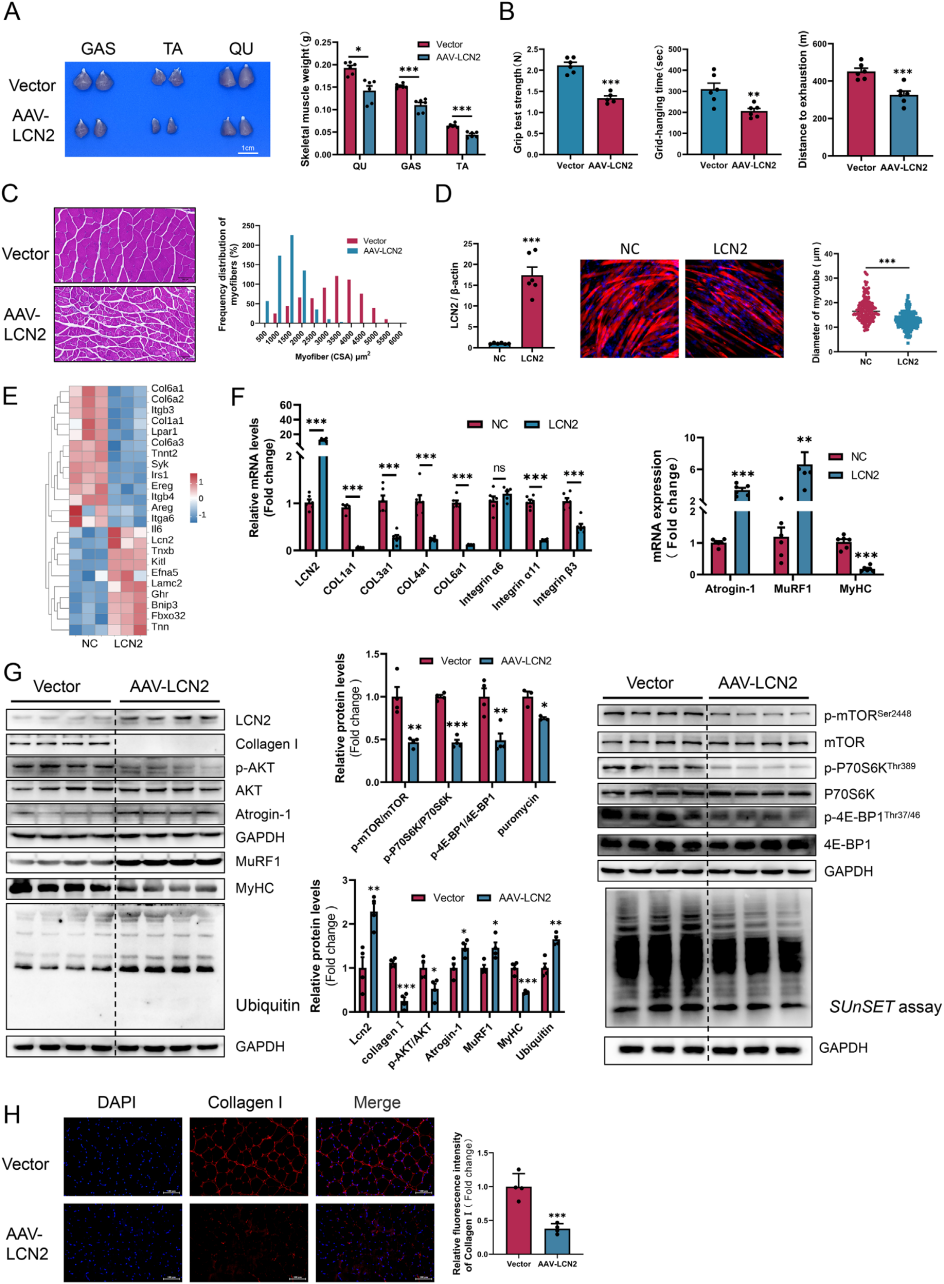
Exogenous LCN2 induces ECM damage and skeletal muscle atrophy in mice. (A) Gross morphology of the GAS, TA, and QU muscles from mice in the AAV9 empty vector control group (Vector) and AAV9‐mediated LCN2 overexpression group (AAV‐LCN2), with quantitative analysis of skeletal muscle wet weight (*n* = 6 per group, scale bar, 1 cm). (B) Quantitative analysis of skeletal muscle function in mice from each group, including the grip strength test, grid‐hanging endurance test, and exhaustive running distance on a treadmill (*n* = 6 per group). (C) H&E staining of gastrocnemius muscle sections from mice in each group, with the frequency distribution of myofiber CSA (Scale bar, 50 µm). (D) Immunofluorescence staining of myosin heavy chain (MyHC, red) in C2C12 myotubes from the negative control (NC) and LCN2 overexpression (LCN2) groups, with nuclei labeled with DAPI (blue). The quantitative violin plot of myotube diameter is shown on the right (Scale bar, 100 µm). (E, F) Heatmap of core differentially expressed genes related to muscle atrophy in C2C12 myotubes (plotted based on row‐normalized Z‐score of TPM values) and quantitative real‐time PCR validation of the relative mRNA levels of core ECM structural genes and muscle atrophy marker genes (*n* = 6 per group). (G) Western blot analysis of LCN2, Collagen I, core proteins of the AKT pathway, muscle‐specific atrophy ubiquitin ligases Atrogin‐1 and MuRF‐1, myofiber marker MyHC, and global protein ubiquitination levels in the GAS of mice from each group. The right panel shows western blot analysis of the phosphorylation levels of core proteins in the mTOR pathway in C2C12 myotubes and the SUNSET puromycin incorporation assay to detect the de novo protein synthesis rate in myotubes. GAPDH was used as the loading control (*n* = 4 per group). (H) Immunofluorescence staining of Collagen I (red) in GAS muscle sections from mice in each group, with nuclei labeled with DAPI (blue). Quantitative analysis of the relative fluorescence intensity of Collagen I is shown on the right (scale bar, 100 µm). All quantitative data are presented as the mean ± standard error of the mean (SEM). ^*^ *p*<0.05, ^**^ *p*<0.01, ^***^ *p*<0.001.

To further define the mechanisms underlying LCN2‐driven muscle atrophy, we performed transcriptome sequencing of LCN2‐overexpressing and negative control (NC) C2C12 myotubes. GO functional enrichment analysis revealed that the extracellular space/region was the most significantly enriched Biological Process (BP) category, with DEGs also overrepresented in biological processes critical for skeletal muscle homeostasis, including cell surface receptor signaling, cellular stress response, and organ development (Figure S1A). Concurrently, KEGG pathway enrichment analysis demonstrated that DEGs were significantly enriched in pathways regulating cellular structural homeostasis, metabolic reprogramming, and the inflammatory stress response (Figure S1B). Focusing on the muscle atrophy‐related core pathways, the ECM‐receptor interaction was the most significantly enriched pathway, whereas the PI3K‐Akt signaling pathway harbored the largest number of enriched DEGs (Figure S1C). Additional enriched pathways included FoxO and mTOR signaling, ubiquitin‐mediated proteolysis, autophagy, and MAPK and AMPK signaling. The core gene expression heatmap further showed that LCN2 overexpression widely downregulated collagen family genes (Col1a1 and Col6a1/2/3) and integrin receptor genes (Itga6 and Itgb3/4), disrupting ECM structure and upstream activation of the PI3K‐Akt pathway. Concurrently, the insulin signaling molecule Irs1 was downregulated and the endogenous AKT inhibitor Trib3 was upregulated, further suppressing PI3K‐Akt pathway activity. Downstream upregulation of the mTOR pathway negative regulators Flcn and Fnip2 indicated that the muscle protein synthesis program was inhibited. FoxO target genes, including the atrophy marker Fbxo32 (Atrogin‐1), core mitophagy gene Bnip3, and positive regulators of FoxO transcriptional activity, Atm and Ccng2, were all significantly upregulated, indicating excessive activation of the muscle protein degradation program (Figure 2E).

We validated these transcriptomic findings via qRT‐PCR, which confirmed that LCN2 overexpression in C2C12 myotubes significantly downregulated mRNA levels of the core collagen genes (Col1a1, Col3a1, Col4a1, Col6a1), integrin mechanotransduction receptors (Itga6, Itgb3), and myotube maturation marker MyHC, while markedly upregulating the mRNA levels of muscle atrophy markers Atrogin‐1 and MuRF1 (Figure 2F). Western blot analysis showed that LCN2 overexpression downregulated type I collagen protein in mouse GAS; inhibited phosphorylation of AKT, mTOR, P70S6K, and 4E‐BP1 to block the PI3K‐Akt‐mTOR axis; upregulated Atrogin‐1 and MuRF1 protein levels; increased global skeletal muscle ubiquitination; and reduced MyHC expression (Figure 2G). The in vitro SUnSET assay further confirmed that LCN2 significantly suppressed global myotube protein synthesis. Immunofluorescence staining verified that LCN2 overexpression markedly disrupted the endomysial type I collagen structure in mouse skeletal muscle (Figure 2H). Taken together, LCN2 induces skeletal muscle atrophy by disrupting ECM homeostasis, inhibiting the PI3K‐Akt‐mTOR anabolic pathway, and activating FoxO‐mediated ubiquitin‐proteasome catabolic signaling, thereby bidirectionally perturbing the muscle protein turnover balance.

### 3.3 GR Targets the Glucocorticoid Response Element Motif to Transcriptionally Activate LCN2

We confirmed that LCN2 is a core mediator of GC‐induced skeletal muscle atrophy. However, the mechanism underlying its upregulation under GC stimulation remains uncharacterized. Therefore, we explored the transcriptional regulatory mechanisms underlying GC‐modulated LCN2 expression. Fully differentiated C2C12 myotubes were treated with Dex combined with the GR‐specific inhibitor RU486. Dex significantly upregulated *Lcn2* mRNA and protein levels, which were effectively blocked by RU486 co‐treatment (Figure 3A,B), indicating that GC‐driven transcriptional activation of LCN2 is strictly dependent on GR activation. JASPAR database analysis of the 2000 bp upstream *Lcn2* promoter region identified a highly conserved GRE predicted as a direct GR‐binding site (Figure 3C). To validate the function of this motif, we constructed dual‐luciferase reporter vectors containing the wild‐type (WT) *Lcn2* promoter and a GRE core sequence site‐directed mutant (MUT). Dex significantly enhanced WT *Lcn2* promoter activity, whereas the GRE core motif mutation completely abolished Dex‐induced transcriptional activation (Figure 3D), confirming that the GRE motif is the core functional region for GR‐mediated LCN2 transcription. We further performed a ChIP assay to verify the endogenous binding between GR and the *Lcn2* promoter: under basal conditions, GR barely bound to the *Lcn2* promoter, while Dex stimulation induced marked enrichment of activated GR at the GRE motif segment, with no non‐specific enrichment in the IgG negative control (Figure 3E). This explicitly confirms that GC‐induced GR nuclear translocation enables direct chromatin‐level binding to the *Lcn2* promoter GRE region. Collectively, these results demonstrate that GC directly initiates LCN2 transcriptional expression via GR activation and targeted binding to the conserved GRE motif in the *Lcn2* promoter.

**FIGURE 3:**
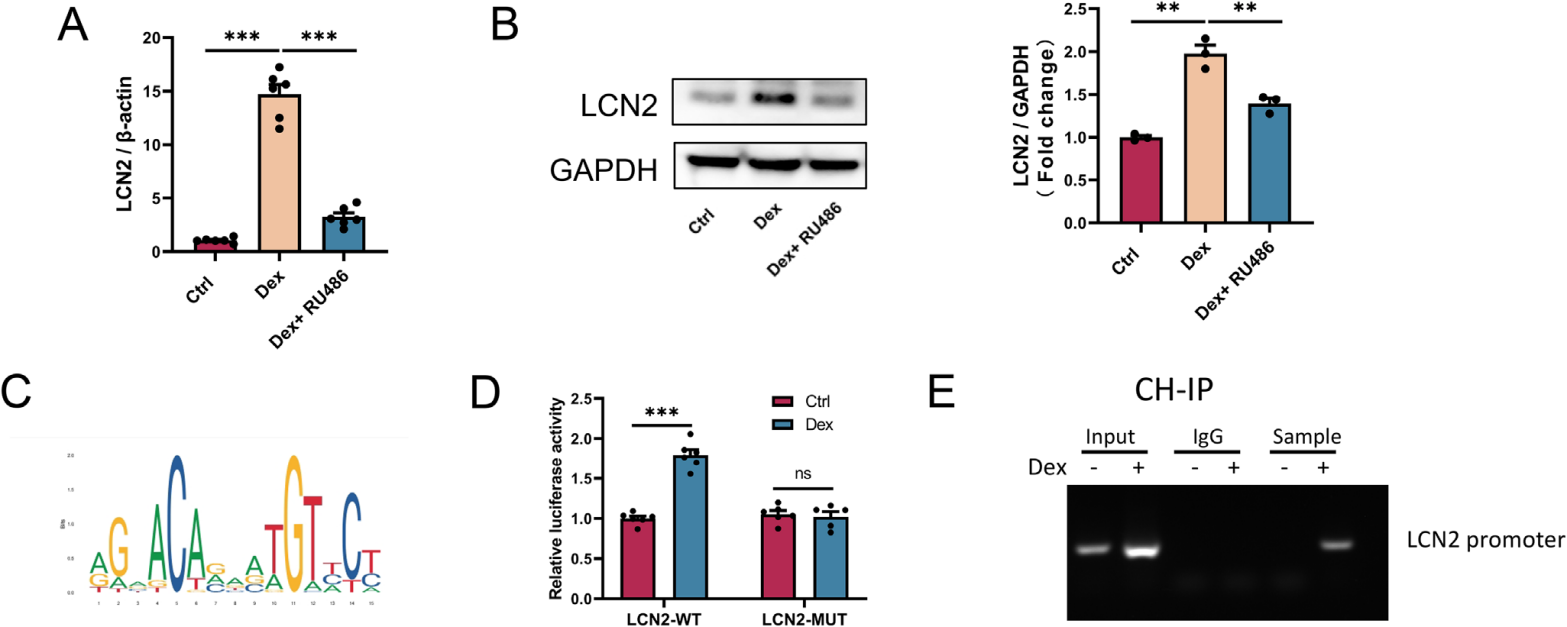
GR Targets the GRE element to transcriptionally activate LCN2. (A) Quantitative real‐time PCR analysis of relative LCN2 mRNA levels in differentiated C2C12 myotubes from the control group (Ctrl), dexamethasone (Dex) treatment group, and Dex plus GR‐specific inhibitor RU486 co‐treatment group (Dex+RU486), with β‐actin as the reference gene (*n* = 6 per group). (B) Western blot analysis of LCN2 protein expression in C2C12 myotubes from each group, with GAPDH as the loading control and corresponding quantitative analysis of protein levels (*n* = 3 per group). (C) Sequence motif of the conserved glucocorticoid response element (GRE) in the LCN2 promoter region predicted using the JASPAR database. (D) Dual‐luciferase reporter assay was used to detect the effect of Dex treatment on the transcriptional activity of the wild‐type LCN2 promoter (LCN2‐WT) and GRE core sequence‐mutated LCN2 promoter (LCN2‐MUT) (*n* = 6 per group). (E) Chromatin immunoprecipitation (ChIP) assay to verify endogenous binding between GR and the GRE region of the LCN2 promoter upon Dex stimulation. Input: total chromatin input control; IgG: isotype antibody‐negative control. All quantitative data are presented as the mean ± standard error of the mean (SEM). ^**^ *p*<0.01, ^***^ *p*<0.001.

### 3.4 Skeletal Muscle‐Specific Silencing of LCN2 Alleviates Glucocorticoid‐Induced Muscle Atrophy

We previously confirmed that LCN2, a direct transcriptional target of GR, drives myotube atrophy by disrupting ECM homeostasis; however, its pathological necessity and targeted therapeutic value in GC‐induced myopathy in vivo remain unclear. To this end, we constructed a skeletal muscle‐specific LCN2 knockdown mouse model via intramuscular injection of HBAAV2/9‐MHCK7‐shLcn2, with non‐targeted scrambled shRNA (shCtrl) as the control and established a steroid myopathy model via intraperitoneal injection of Dex to systematically evaluate the protective effect of LCN2 silencing on skeletal muscle. Under basal physiological conditions, skeletal muscle‐specific LCN2 silencing had no significant effects on gross morphology, wet weight, myofiber CSA, and basal motor function of the QU, GAS, and TA in mice (Figure 4A–C). Dex intervention induced significant skeletal muscle atrophy in shCtrl mice, manifested as reduced volume and wet weight of multiple muscle groups; impaired grip strength, grid hanging endurance, and treadmill exhaustion distance; and markedly decreased myofiber CSA, whereas skeletal muscle‐specific LCN2 silencing markedly reversed all these Dex‐induced pathological changes, effectively alleviating muscle mass loss, functional decline, and myofiber atrophy (Figure 4A–C). Collectively, targeted silencing of skeletal muscle LCN2 effectively alleviated Dex‐induced muscle atrophy and functional decline without disturbing the physiological homeostasis of normal skeletal muscles.

**FIGURE 4:**
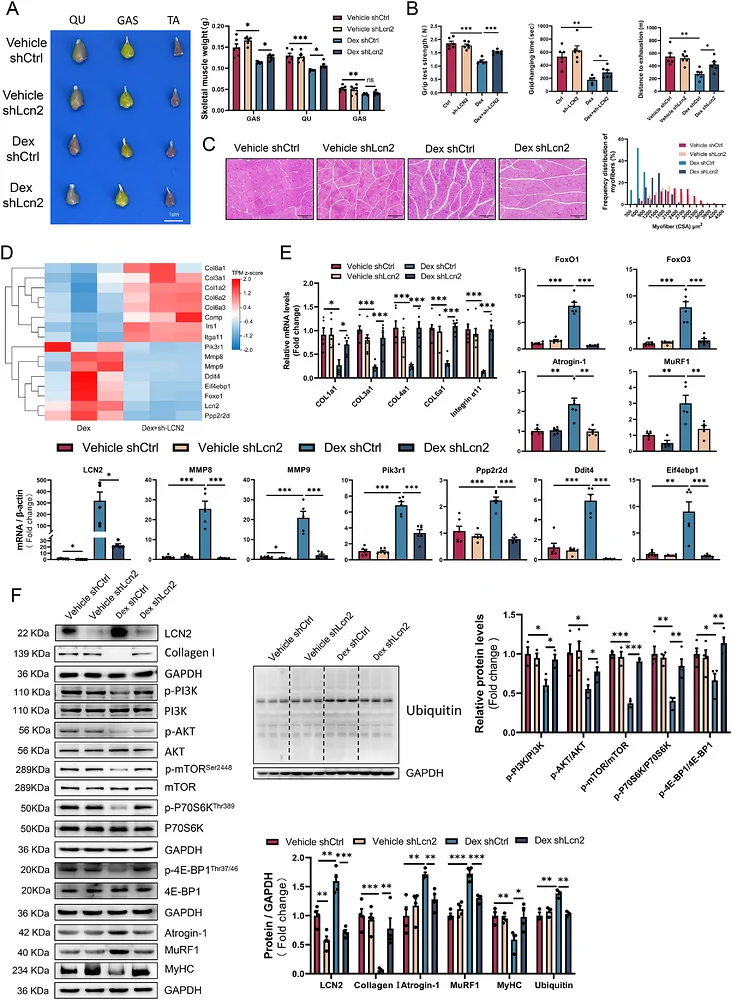
Skeletal muscle‐specific silencing of LCN2 alleviates glucocorticoid‐induced muscle atrophy. (A) Quantitative analysis of skeletal muscle wet weight in the QU, GAS, and TA of mice in each group. Groups: Vehicle shCtrl (empty control virus group), Vehicle shLcn2 (skeletal muscle‐specific LCN2 knockdown group), Dex shCtrl (dexamethasone‐treated empty control group), Dex shLcn2 (dexamethasone‐treated LCN2 knockdown group) (*n* = 6 per group, scale bar, 1 cm). (B) Quantitative analysis of skeletal muscle function in mice from each group, including the grip strength test, grid‐hanging endurance test, and exhaustive running distance on a treadmill (*n* = 6 per group). (C) H&E staining of GAS sections from mice in each group, with the frequency distribution of myofiber CSA (scale bar, 100 µm). (D) Heatmap of core differentially expressed genes related to muscle atrophy in the GAS from the Dex shCtrl and Dex shLcn2 groups plotted based on row‐normalized Z‐score of TPM values (*n* = 3 per group). (E) Quantitative real‐time PCR validation of the relative mRNA levels of core ECM structural genes, LCN2, ECM‐degrading enzymes, regulatory factors of the PI3K‐Akt pathway, inhibitory molecules of the mTOR pathway, core transcription factors, and marker genes of muscle atrophy in the GAS from each group, with β‐actin as the reference gene (*n* = 6 per group). (F) Western blot analysis of LCN2, Collagen I, total and phosphorylated core proteins of the PI3K‐Akt‐mTOR pathway, muscle‐specific atrophy ubiquitin ligases Atrogin‐1 and MuRF‐1, myofiber maturation marker MyHC, and global protein ubiquitination levels in the GAS from each group, with a corresponding quantitative analysis of protein levels. GAPDH was used as the loading control (*n* = 3‐4 per group). All quantitative data are presented as the mean ± standard error of the mean (SEM). ^*^ *p*<0.05, ^**^ *p*<0.01, ^***^ *p*<0.001.

To elucidate the downstream molecular mechanism underlying the alleviation of GC‐induced muscle atrophy by skeletal muscle‐specific LCN2 silencing, we performed transcriptome sequencing of GAS tissues from the Dex‐treated shCtrl control group (Dex group) and shLcn2 knockdown group (Dex_sh_LCN2 group) to systematically characterize the transcriptomic reprogramming profile mediated by LCN2 silencing. Screening of DEGs showed that compared with the Dex group, 735 genes were significantly differentially expressed in the Dex_sh_LCN2 group, including 195 upregulated and 540 downregulated genes (Figure S2A). GO Cellular Component (CC) enrichment analysis revealed that the most significantly enriched terms for DEGs were all centered on the regulation of skeletal muscle ECM homeostasis, including extracellular region, extracellular space, collagen trimer, collagen‐containing extracellular matrix, and extracellular matrix. Meanwhile, DEGs were also significantly enriched in cellular components closely related to mechanical signal transmembrane transduction, such as the external side of the plasma membrane, plasma membrane raft, and integrin αMβ2 complex (Figure S2B). This result was inversely correlated with the enrichment results of our previous LCN2 overexpression experiment, which directly confirmed that the core protective effect of targeted LCN2 silencing was to reverse GC‐induced structural damage and homeostasis imbalance of the skeletal muscle ECM. To focus on the core biological mechanism related to muscle atrophy, we screened and visualized the core pathways closely related to skeletal muscle growth and development, protein metabolic homeostasis, and the pathological progression of muscle atrophy from all significantly enriched pathways (Q value < 0.05) (Figure S2C). The results demonstrated that the PI3K‐Akt signaling pathway served as the core regulatory hub in this enrichment analysis, with the greatest number of mapped DEGs and the strongest enrichment significance. Meanwhile, the insulin signaling, mTOR signaling, and focal adhesion pathways that directly modulate skeletal muscle protein anabolism; the FoxO signaling (Figure S2D,E), ubiquitin‐mediated proteolysis, and autophagy pathways that govern muscle catabolism; and the ECM‐receptor interaction pathway were all significantly enriched. In addition, inflammatory pathways related to the pathological amplification effect of muscle atrophy (IL‐17 and TNF signaling pathways) and pathways related to cellular stress and death (ferroptosis and apoptosis) were also markedly enriched. The heatmap of core functional gene expression showed that the core ECM structural components (Col8a1, Col3a1, Col1a2, Col6a2, Col6a3) and upstream activating molecules of the insulin‐PI3K‐Akt pathway (Irs1, Itga11), which were significantly downregulated after Dex treatment, were markedly restored after skeletal muscle LCN2 silencing; while the ECM‐degrading enzymes Mmp8/Mmp9, endogenous inhibitors of the mTOR pathway Ddit4/Eif4ebp1, core muscle atrophy transcription factor Foxo1, and LCN2 itself, which were significantly upregulated after Dex treatment, were markedly downregulated after LCN2 silencing (Figure 4D).

To validate the transcriptome results, we detected the transcriptional levels of core DEGs via qRT‐PCR. Dex treatment significantly downregulated the mRNA levels of core collagen family genes and integrin mechanotransduction receptor Itga11, whereas LCN2 silencing significantly reversed this expression inhibition of ECM‐related genes; meanwhile Dex‐induced aberrant upregulation of Lcn2, Mmp8/Mmp9, PI3K‐Akt pathway negative regulators, mTOR pathway inhibitors, Foxo1/Foxo3, and atrophy markers Atrogin‐1 and MuRF1 were all significantly reversed by LCN2 silencing, consistent with the transcriptome data (Figure 4E). Western blot further confirmed that LCN2 silencing completely reversed Dex‐induced LCN2 upregulation and type I collagen downregulation; the reduced phosphorylation of PI3K, AKT, mTOR, and their downstream effectors P70S6K and 4E‐BP1 induced by Dex were significantly restored by LCN2 knockdown, verifying reactivation of the PI3K‐Akt‐mTOR anabolic pathway. Correspondingly, Dex‐induced upregulation of Atrogin‐1 and MuRF1, MyHC downregulation, and elevated global protein ubiquitination were all markedly reversed by LCN2 silencing, confirming that LCN2 knockdown simultaneously inhibited excessive protein degradation and bidirectionally restored skeletal muscle protein metabolic balance (Figure 4F). Given that oral administration is the most common route for long‐term clinical glucocorticoid therapy, we established a Dex‐induced muscle atrophy mouse model via oral gavage to verify whether silencing of LCN2 could exert a comparable protective effect under this clinically relevant dosing regimen (Figure S3). The intragastric Dex challenge induced significant muscle atrophy in mice, including reduced wet weight in multiple muscle groups, impaired grip strength and exercise endurance, and decreased myofiber cross‐sectional area. Skeletal muscle‐specific LCN2 silencing effectively ameliorated all atrophic phenotypes and preserved muscle mass and function (Figure S3A–C). Molecular analyses yielded results that were highly consistent with those of the intraperitoneal injection model. LCN2 knockdown efficiently reversed Dex‐induced LCN2 upregulation and ECM collagen reduction, restored phosphorylation of the AKT‐mTOR anabolic cascade, concurrently suppressed the overexpression of muscle atrophy markers Atrogin‐1 and MuRF1, and elevated global protein ubiquitination (Figure S3D–F). Collectively, targeted silencing of LCN2 reversed Dex‐induced muscle metabolic dysregulation and ameliorated muscle atrophy by restoring ECM homeostasis, reactivating the PI3K‐Akt‐mTOR signaling pathway, and correcting skeletal muscle protein metabolic imbalance.

### 3.5 Targeted Knockdown of LCN2 Alleviates Dexamethasone‐Induced Myotube Atrophy

We validated this in vitro assay using fully differentiated C2C12 myotubes. We knocked down LCN2 via siRNA transient transfection, established a Dex‐induced myotube atrophy model, and evaluated the protective effect of LCN2 knockdown against myotube atrophy. MyHC immunofluorescence staining and diameter quantification revealed that Dex induced significant myotube atrophy and reduced myotube diameter, which were markedly attenuated by LCN2 knockdown, with partial restoration of normal myotube morphology (Figure 5A). Western blot results were fully consistent with our in vivo data: Dex significantly upregulated LCN2, Atrogin‐1 and MuRF1 in C2C12 myotubes, and downregulated mature myotube core marker MyHC; all these Dex‐induced aberrant protein changes were markedly mitigated by LCN2 knockdown (Figure 5B). Further mechanistic validation showed that Dex significantly inhibited mTOR pathway activation, as indicated by reduced phosphorylation of mTOR and its downstream effectors P70S6K and 4E‐BP1, which was significantly alleviated by LCN2 knockdown (Figure 5C). The in vitro SUnSET puromycin incorporation assay further confirmed that LCN2 knockdown significantly restored the Dex‐induced suppression of myotube protein synthesis, mitigating atrophy by rescuing the anabolic program (Figure 5D).

**FIGURE 5:**
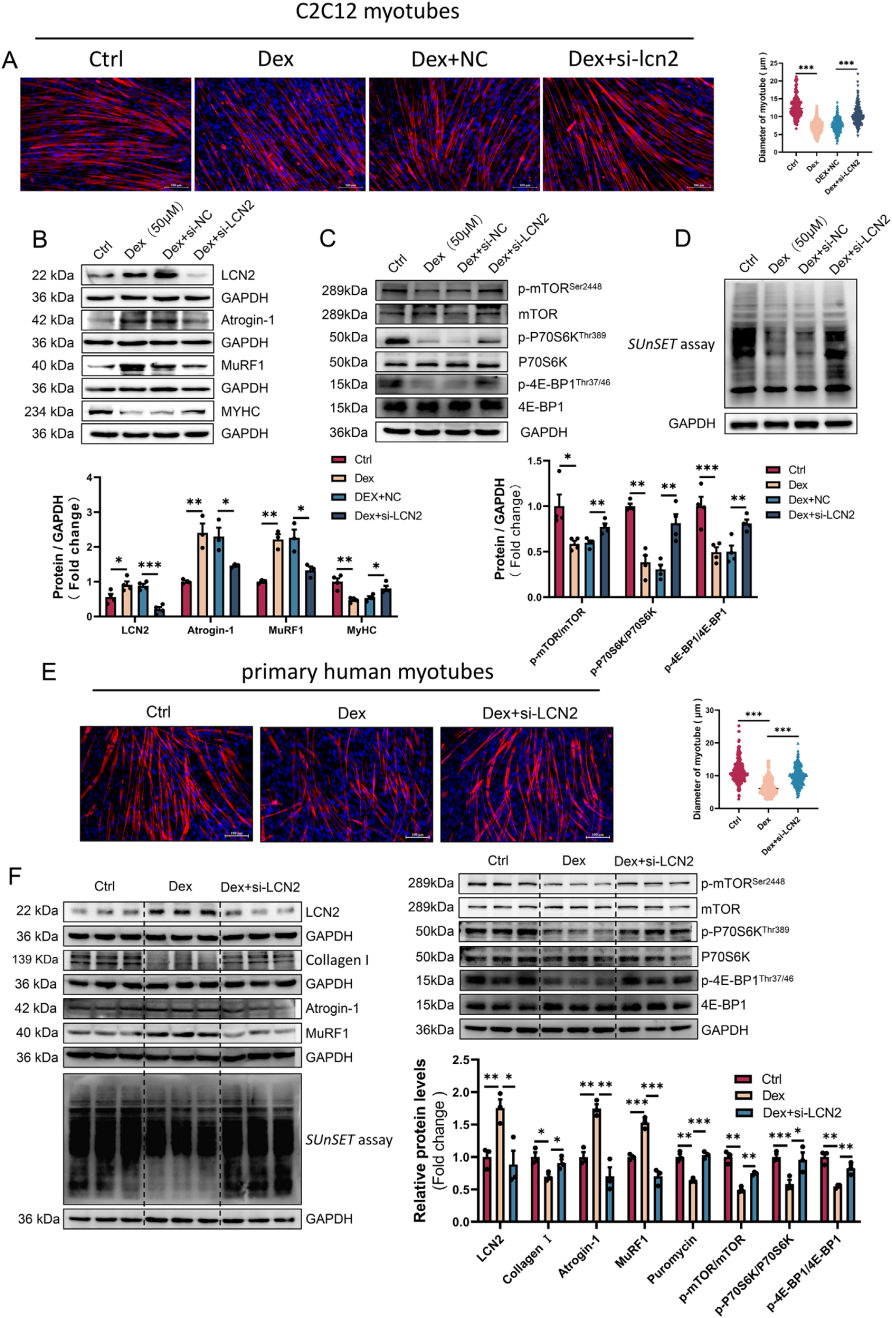
Targeted knockdown of LCN2 alleviates dexamethasone‐induced myotube atrophy. (A) Immunofluorescence staining of MyHC in differentiated C2C12 myotubes from each group (MyHC in red labels mature myotubes; nuclei were labeled with DAPI in blue), with the corresponding quantitative violin plot of myotube diameter. Groups: control (Ctrl), 50 µm dexamethasone treatment (Dex), Dex plus negative control siRNA transfection (Dex+NC), Dex plus LCN2‐targeting siRNA transfection (Dex+si‐LCN2) (Scale bar, 50 µm). (B) Western blot analysis of LCN2, muscle‐specific atrophy ubiquitin ligases Atrogin‐1 and MuRF‐1, and myotube maturation marker MyHC in C2C12 myotubes from each group, with corresponding quantitative analysis. GAPDH was used as the loading control (*n* = 3‐4 per group). (C) Western blot analysis of total and phosphorylated core proteins of the mTOR signaling pathway (mTOR, P70S6K, and 4E‐BP1) in C2C12 myotubes from each group, with quantitative analysis of the phosphorylated/total protein ratio. GAPDH was used as the loading control (*n* = 4 per group). (D) SUNSET puromycin incorporation assay to detect the de novo protein synthesis rate in C2C12 myotubes from each group. GAPDH was used as the loading control. (E) Immunofluorescence staining of MyHC in differentiated human primary myotubes from each group (MyHC in red labels mature myotubes; nuclei were labeled with DAPI in blue), with the corresponding quantitative violin plot of the myotube diameter. Groups: control (Ctrl), 50 µM dexamethasone treatment (Dex), Dex plus LCN2‐targeting siRNA transfection (Dex+si‐LCN2) (Scale bar, 100 µm). (F) Western blot analysis of LCN2, Collagen I, muscle‐specific atrophy ubiquitin ligases Atrogin‐1 and MuRF1, total and phosphorylated core proteins of the mTOR signaling pathway (mTOR, P70S6K, and 4E‐BP1), and de novo protein synthesis detected by the SUnSET puromycin incorporation assay in human primary myotubes from each group, with corresponding quantitative analysis of total protein levels and phosphorylated/total protein ratios. GAPDH was used as the loading control (*n* = 3 per group). All quantitative data are presented as the mean ± standard error of the mean (SEM). ^*^ *p*<0.05, ^**^ *p*<0.01, ^***^ *p*<0.001.

To confirm the evolutionary conservation and clinical translational relevance of these findings, we performed parallel validation in differentiated human primary myotubes. Consistent with the results in C2C12 myotubes, LCN2 knockdown significantly alleviated Dex‐induced myotube atrophy and restored myotube caliber (Figure 5E). Mechanistically, LCN2 silencing markedly reversed Dex‐induced upregulation of LCN2 and the muscle‐specific E3 ligases Atrogin‐1 and MuRF1 and rescued the loss of type I collagen. Furthermore, LCN2 knockdown restored the phosphorylation levels of mTOR and its downstream effectors, P70S6K and 4E‐BP1, indicating reactivation of the anabolic signaling cascade. The SUnSET puromycin incorporation assay further confirmed that LCN2 depletion significantly ameliorated Dex‐induced suppression of global protein synthesis in human primary myotubes (Figure 5F).

Collectively, these in vitro findings are consistent with our in vivo results, demonstrating that LCN2 knockdown bidirectionally ameliorates GC‐induced myotube protein homeostasis imbalance via both anabolic and catabolic pathways, ultimately alleviating Dex‐induced myotube atrophy.

### 3.6 LCN2 Disrupts Skeletal Muscle ECM Homeostasis Via Specific Binding to MMP9

MMPs are key effector proteases that mediate collagen fibril degradation and ECM structural remodeling in the skeletal muscle microenvironment [21]. Our study established that targeted silencing of LCN2 attenuated the aberrant upregulation of both MMP8 and MMP9. We hypothesized that LCN2 drives skeletal muscle atrophy via MMP‐dependent ECM disruption and therefore investigated the direct protein‐protein interactions between LCN2 and MMP8/MMP9. Co‐IP assay results revealed that in C2C12 myotubes, LCN2 overexpression significantly enhanced the protein expression of both MMP8 and MMP9; in the immunoprecipitates of LCN2, specific binding of LCN2 to MMP9 was detected, whereas no specific enrichment of MMP8 was observed (Figure 6A). Immunofluorescence colocalization assay further confirmed that LCN2 and MMP9 displayed obvious intracellular colocalization both under physiological conditions and in the Dex‐induced myotube atrophy model, and the colocalization signal was enhanced along with the upregulated LCN2 expression after Dex treatment; in contrast, no evident spatial colocalization was found between LCN2 and MMP8 (Figure 6B). Collectively, these results demonstrate that LCN2 regulates collagen degradation and ECM remodeling through specific protein‐protein interaction with MMP9 rather than MMP8.

**FIGURE 6:**
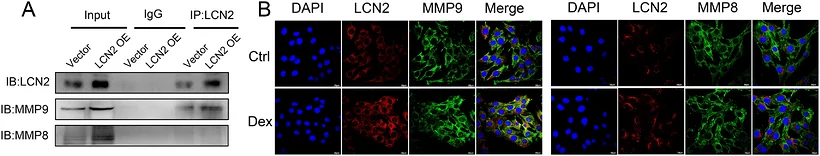
LCN2 disrupts skeletal muscle ECM homeostasis via specific binding to MMP9. (A) Co‐immunoprecipitation (Co‐IP) assay to detect the endogenous physical interaction between LCN2 and MMP9/MMP8 in C2C12 myotubes from the empty vector control (Vector) and LCN2 overexpression group (LCN2 OE) groups. Input: Total cell protein loading control; IgG: isotype antibody negative control; IB: immunoblotting antibody. (B) Immunofluorescence co‐localization staining to detect the intracellular spatial co‐localization of LCN2 with MMP9 or MMP8 in C2C12 myotubes from the control (Ctrl) and dexamethasone (Dex)‐induced atrophy model groups. LCN2 was labeled with red fluorescence, MMP9/MMP8 were labeled with green fluorescence, and nuclei were labeled with DAPI (blue fluorescence) (Scale bar: 20 µm).

### 3.7 MMP9 Rescue Abrogates the Protective Effect of LCN2 Silencing Against Glucocorticoid‐Induced Skeletal Muscle Atrophy

To clarify whether MMP9 is the essential downstream effector of LCN2‐induced muscle atrophy, we performed an in vivo rescue assay by replenishing recombinant MMP9 in Dex‐induced muscle atrophy mice with skeletal muscle‐specific LCN2 silencing to evaluate its impact on the protective effect of LCN2 knockdown. The results showed that MMP9 replenishment significantly blunted the protective effect of LCN2 silencing against muscle atrophy, leading to overt re‐atrophy of mouse QU, GAS, and TA, with significantly reduced skeletal muscle wet weight and muscle weight/body weight ratio compared with those of the Dex+shLcn2 group (Figure 7A). Meanwhile, MMP9 replenishment markedly decreased grip strength, grid hanging endurance, and treadmill exhaustion distance, and the ameliorative effect of LCN2 silencing on skeletal muscle function was significantly attenuated (Figure 7B). Histological results revealed that MMP9 replenishment significantly reversed the mitigation of myofiber atrophy by LCN2 silencing, as evidenced by a reoccurrence of significant reduction in CSA and a marked increase in the proportion of small‐diameter myofibers (Figure 7C). Mechanistic analysis further delineated the molecular cascade of the LCN2‐driven pro‐atrophic effect mediated by MMP9: MMP9 replenishment re‐disrupted skeletal muscle ECM homeostasis via its collagenolytic activity (Figure 7D), reversing the restoration of core ECM structural proteins, including type I collagen, by LCN2 silencing. This, in turn, inhibited integrin‐mediated mechanotransduction, significantly downregulated the phosphorylation and activation of focal adhesion kinase (FAK, the core hub of mechanotransduction), and synchronously suppressed the physiological activation of its downstream PI3K‐Akt‐mTOR signaling axis, thereby re‐blocking protein synthesis in skeletal muscle. Meanwhile, MMP9 replenishment‐induced Akt pathway inhibition markedly decreased FoxO3 phosphorylation, further driving the upregulation of the muscle‐specific atrophy ubiquitin ligases Atrogin‐1 and MuRF1. This ultimately led to hyperactivation of the ubiquitin‐proteasome system, elevated global protein ubiquitination in skeletal muscle, and reinitiation of the muscle protein degradation program (Figure 7E). Taken together, this in vivo rescue experiment confirms that MMP9 is the essential downstream effector of LCN2‐mediated GC‐induced skeletal muscle atrophy.

**FIGURE 7:**
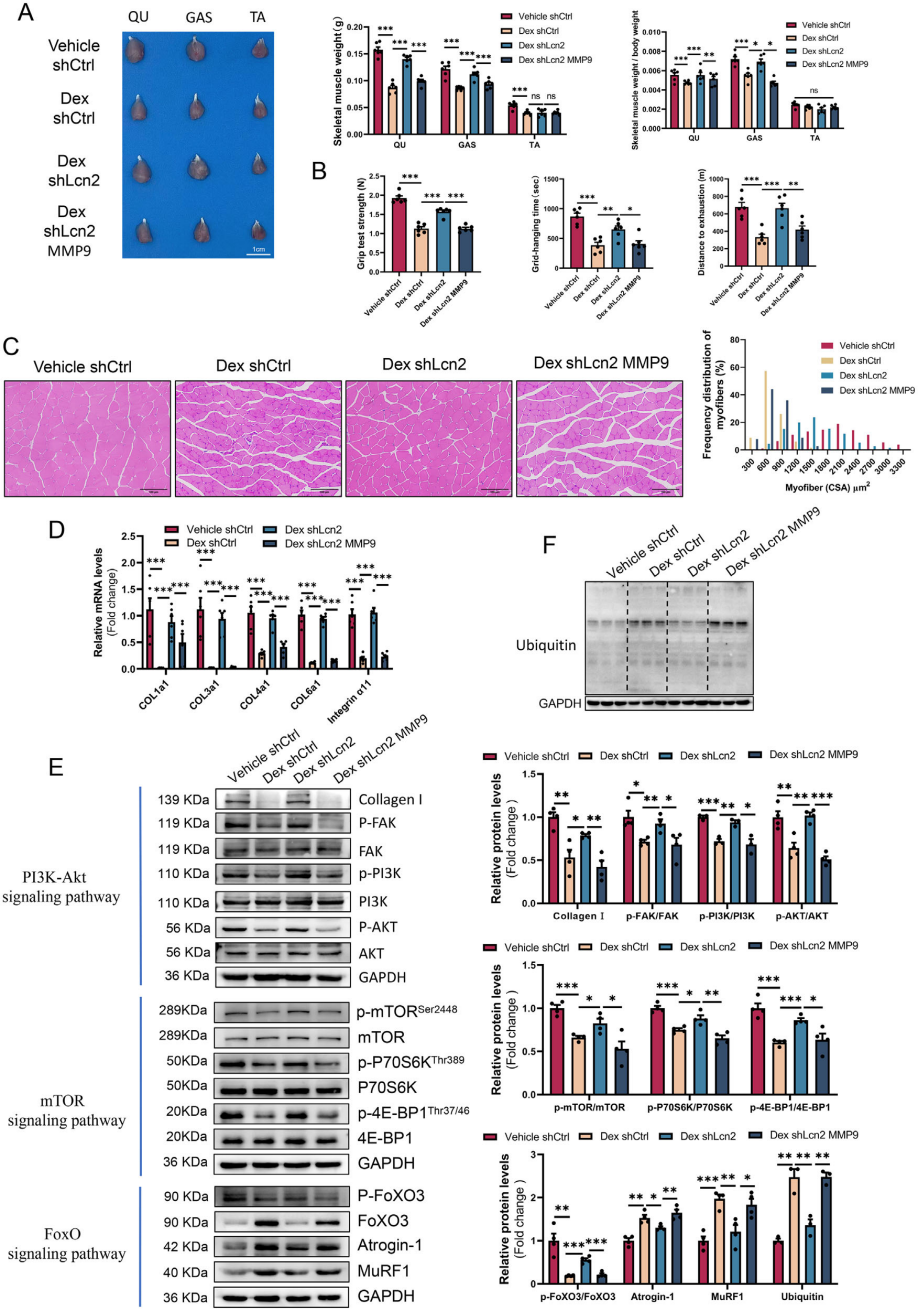
MMP9 rescue abrogates the protective effect of LCN2 silencing against glucocorticoid‐induced skeletal muscle atrophy. (A) Quantitative analysis of skeletal muscle wet weight and muscle wet weight/body weight ratio in the QU, GAS, and TA of mice in each group. Groups: Vehicle shCtrl (empty control virus group), Dex shCtrl (dexamethasone‐treated empty control group), Dex shLcn2 (dexamethasone‐treated skeletal muscle‐specific LCN2 knockdown group), Dex shLcn2 MMP9 (dexamethasone‐treated LCN2 knockdown with MMP9 overexpression rescue group). (*n* = 6 per group, Scale bar, 1 cm). (B) Quantitative analysis of skeletal muscle function in mice from each group, including the grip strength test, grid‐hanging endurance test, and exhaustive running distance on a treadmill (*n* = 6 per group). (C) H&E staining of gastrocnemius muscle sections from mice in each group, with the frequency distribution of myofiber CSA (Scale bar, 50 µm). (D) Quantitative real‐time PCR validation of the relative mRNA levels of core ECM structural genes (Col1a1, Col3a1, Col4a1, Col6a1) and integrin mechanoreceptor gene Integrin α11 in gastrocnemius muscles from each group, with β‐actin as the reference gene (*n* = 6 per group). (E) Western blot analysis of Collagen I, total and phosphorylated core proteins of the FAK‐PI3K‐Akt‐mTOR and FoxO3 pathways, and muscle‐specific atrophy ubiquitin ligases, Atrogin‐1 and MuRF‐1, in the GAS from each group, with corresponding quantitative analysis. GAPDH was used as the loading control (*n* = 4 per group). (F) Western blot analysis of global protein ubiquitination levels in the GAS from each group, with corresponding quantitative analysis. GAPDH was used as the loading control (*n* = 3 per group). All quantitative data are presented as the mean ± standard error of the mean (SEM). ^*^ *p*<0.05, ^**^ *p*<0.01, ^***^ *p*<0.001.

## 4 Discussion

GC‐induced skeletal muscle atrophy is the most prevalent clinical iatrogenic myopathy, which impairs motor function and quality of life of patients and significantly increases the risk of falls, fractures, and mortality [22]. Canonical mechanisms driving this pathology center on FoxO‐mediated UPS hyperactivation and inhibition of Akt/mTOR‐dependent protein anabolism [23]. Although GC‐mediated GR activation is a well‐established upstream initiating event, the critical downstream effectors that directly execute GR‐driven muscle atrophy remain incompletely characterized, and clinically effective targeted interventions are still lacking. In this study, we identified LCN2 as a direct transcriptional target and systematically demonstrated that LCN2 is a core driver of GC‐induced skeletal muscle atrophy. Mechanistically, GC‐activated LCN2 directly binds to MMP9 to trigger ECM homeostasis imbalance, which blocks integrin‐focal adhesion‐mediated mechanotransduction and ultimately drives the bidirectional dysregulation of skeletal muscle protein metabolism via the PI3K‐Akt‐mTOR/FoxO cascade.

As a multifunctional secreted glycoprotein, LCN2 has been established as a key negative regulator of skeletal muscle pathophysiology in multiple disease contexts, including obesity‐related and cancer cachexia‐associated sarcopenia [16, 17, 18, 19]. Notably, previous studies have reported conflicting roles of LCN2 in skeletal muscle disorders, with LCN2 deficiency reportedly impairing satellite cell activation and muscle regeneration [24]. We postulate that this functional discrepancy stems from the context‐dependent effects of LCN2 across distinct pathological states. In this study, we demonstrated for the first time that GC directly transcriptionally activates LCN2 expression in skeletal muscle via GR binding to the conserved GRE in the *Lcn2* promoter, and the secreted LCN2 exerts its biological functions extracellularly, which further complements the downstream transcriptional regulatory network of GR in skeletal muscle. Previous studies have shown that members of the Krüppel‐like factor (KLF) family (such as KLF9 and KLF15) are direct targets of GR, which mediate GC‐induced muscle atrophy by regulating the transcription of muscle atrophy‐related genes [25, 26]. In contrast, our study identified LCN2 as a novel downstream effector of GR, which drives muscle atrophy through a previously unrecognized mechanism centered on the regulation of ECM remodeling, rather than direct transcriptional control of atrophy‐related genes.

Current therapeutic strategies for GC‐induced muscle atrophy range from conservative supportive measures to targeted molecular therapies. Nutritional supplementation with proteins, minerals, and selected natural extracts yields mild functional improvement and partial protection against muscle wasting but is generally insufficient to reverse established moderate‐to‐severe atrophy [27, 28, 29, 30]. Exercise supports muscle mass maintenance through systemic biological benefits [31], yet is infeasible for bedridden patients, severely ill populations, and frail older adults. For targeted pharmacological strategies, selective FoxO1 inhibitors represent a promising direction; they suppress MuRF1 induction, enhance protein synthesis via p70S6k phosphorylation, and preserve fast‐twitch fibers [32]. However, specific agents targeting the FoxO pathway remain scarce, and their development faces substantial challenges in target selectivity and in vivo efficacy. Myostatin is a key mediator of GC‐induced muscle atrophy. Diverse myostatin‐blocking agents, including full/fragment antibodies, soluble decoy ActRII receptors, propeptides, natural compounds, and the endogenous antagonist follistatin, have shown robust preclinical efficacy in attenuating atrophy caused by chronic diseases, genetic disorders, and prolonged GC exposure [33, 34]. Nevertheless, poor target specificity remains a core translational bottleneck: cross‐reactivity with other TGF‐β superfamily members such as BMP9 and BMP10 may induce musculoskeletal deformities, limiting clinical benefits [35]. Notably, nearly all of the above strategies focus on the intracellular protein metabolism machinery within mature myofibers, while the skeletal muscle ECM microenvironment, which is a critical upstream regulator of mechanotransduction, cellular signaling, and tissue homeostasis, has long been overlooked in the context of GC‐induced myopathy. Unlike conventional strategies that solely target protein turnover, restoring the structural integrity of the ECM not only increases muscle mass but also rebuilds the mechanical support and microenvironment required for normal myofiber function. This approach offers great promise for restoring muscle contractility and addresses the key limitation documented in prior clinical trials; that is, gains in muscle mass rarely translate into commensurate functional improvements.

Accumulating studies in the tissue regeneration field have consistently demonstrated that tissue microenvironment homeostasis is a core determinant of tissue functional maintenance and injury repair [36, 37, 38]. Disruption of this homeostasis directly triggers cellular dysfunction and pathological deterioration across multiple tissue systems. Skeletal muscle ECM serves as both mechanical support for myofibers and a critical microenvironment that regulates cell signaling and tissue homeostasis [39, 40, 41], with ECM disruption directly driving muscle dysfunction and regenerative impairment. Our transcriptomic data revealed that ECM components and the ECM‐receptor interaction pathway were the most significantly enriched signatures in LCN2‐overexpressing myotubes, with widespread downregulation of core collagen genes and integrin mechanoreceptors, identifying ECM homeostasis imbalance as a key driver of LCN2‐mediated muscle atrophy. Consistently, skeletal muscle‐specific LCN2 knockdown significantly mitigated Dex‐induced dysregulation of ECM‐related genes. We further demonstrated that LCN2 specifically binds to MMP9 (but not MMP8) to enhance its collagenolytic activity, leading to excessive degradation of skeletal muscle ECM components. Critically, in vivo recombinant MMP9 replenishment significantly blunted the protective effect of LCN2 knockdown against GC‐induced muscle atrophy, providing causal evidence for the LCN2‐MMP9 axis in this pathology.

The PI3K‐Akt‐mTOR axis is the core hub governing skeletal muscle protein homeostasis [42]. Here, we found that LCN2‐MMP9 axis‐mediated ECM disruption directly inhibits integrin‐focal adhesion‐mediated transmembrane mechanotransduction, thereby systemically suppressing PI3K‐Akt‐mTOR axis activation in a growth factor‐independent manner. Consequently, bidirectional protein metabolism imbalance characterized by impaired protein synthesis and excessive FoxO‐UPS‐dependent myofibrillar protein degradation was set in motion. Consistent with this process, skeletal muscle‐specific LCN2 knockdown restored Dex‐induced impairment of this mechanotransduction cascade, whereas recombinant MMP9 replenishment blocked the pathway and abrogated the protective effect of LCN2 silencing.

This study had several limitations. First, our assessment of muscle performance was based on in vivo behavioral assays, including grip strength, hanging endurance, and exhaustive running tests. Although these readouts reliably reflect systemic muscle function in living animals, they cannot fully rule out potential contributions from non‐muscle factors, such as hormonal regulation, neural input, and neuromuscular junction integrity, to the observed functional improvements. As isolated muscle contractility experiments were not performed in the current study, we cannot definitively attribute the rescued muscle performance exclusively to the direct cell‐autonomous effects of LCN2 modulation in mature myofibers. Ex vivo functional validation using isolated muscle strips is important in future studies to corroborate the direct role of LCN2 in skeletal muscle contractility. Second, this study focused predominantly on the direct regulatory effects of LCN2 on protein homeostasis in mature myofibers and did not explore the impact of LCN2‐mediated extracellular matrix dysregulation on skeletal muscle satellite cell function. Given that satellite cell dysfunction serves as a key synergistic driver of the progression of muscle atrophy, the mechanistic crosstalk between the LCN2‐ECM regulatory axis and satellite cell activity merits further investigation. Nonetheless, this study systematically elucidated the core mechanism by which LCN2, a direct GR transcriptional target, mediates GC‐induced muscle atrophy via the LCN2‐MMP9‐ECM‐integrin‐Akt/mTOR axis. Our findings not only expand the GR downstream regulatory network in muscle atrophy but also establish a mechanistic link between skeletal muscle ECM microenvironment dysregulation and canonical intracellular atrophy signaling, providing a promising therapeutic target for GC‐induced myopathy. From a translational perspective, targeting the LCN2–MMP9 axis may offer a potential strategy for preserving ECM integrity and preventing glucocorticoid‐associated muscle wasting in patients requiring long‐term GC therapy. Additionally, LCN2 may serve as a potential biomarker for early diagnosis and disease monitoring.

## Author Contributions

Conceptualization, H.S., Y.D., J.W. and H.W.; software, W.X., H.S., Y.D., and Z.L.; methodology, H.S., F.L., X.H. and Y.Z.; formal analysis, Y.D., W.X. and Y.Y.; resources, J.L., X.L. and H.W.; investigation, Y.Y., W.X. and Z.L.; data curation, H.S., Z.L. and X.H.; Writing – original draft preparation, H.S., F.L. and Y.Z.; Writing – review and editing, X.H., J.L. and J.W.; Validation, H.S., X.H. and J.W; visualization, H.S., X.L. and Y.Y; supervision, Y.D. H.W. and J.W; project administration, H.W.; funding acquisition, H.W., Y.Y., and X.L. All authors have read and agreed to the published version of the manuscript.

## Conflicts of Interest

The authors declare no conflicts of interest.

## Supporting information




**Supporting File**: advs77189‐sup‐0001‐SuppMat.doc.

## Data Availability

The data that support the findings of the research are available from the corresponding author upon reasonable request.
