# Supplementary material for: Loss of LCN2 Function Ameliorates Glucocorticoid‐Induced Muscle Atrophy via Remodeling the Extracellular Matrix
Source: Adv Sci (Weinh). 2026 Aug 18;13(62):e77189. doi: 10.1002/advs.77189 (PMC13483132; doi:10.1002/advs.77189)
Supplement: Supplementary file 1 [file ADVS-13-e77189-s001.doc]

**Supplementary information includes:**

Supplementary Figs. S1–3

Tables S1–S2

**Figure S1. GO and KEGG enrichment analysis of DEGs in LCN2-overexpressing C2C12 myotubes.** (A) GO functional enrichment analysis of DEGs. (B) Full-scale KEGG pathway enrichment analysis of DEGs. (C) Focused KEGG enrichment analysis of muscle atrophy-related core pathways. The horizontal axis represents Gene Ratio, dot size represents the number of enriched genes, and the color gradient represents enrichment significance (-log₁₀(Q value)).

**Figure S2. Transcriptome enrichment analysis of GAS tissues from Dex-treated mice with skeletal muscle-specific LCN2 silencing.** (A) Statistical bar plot of DEGs between the Dex group and Dex-sh-LCN2 group. (B) GO Cellular Component enrichment bubble plot of DEGs. (C) KEGG pathway enrichment bubble plot of DEGs. The horizontal axis represents Rich Ratio, dot size represents the number of enriched genes, and the color gradient represents enrichment significance (Q value)

**Figure S3. Skeletal Muscle-Specific Silencing of LCN2 Alleviates Glucocorticoid-Induced Muscle Atrophy.** (A) QU, GAS, and TA muscles from mice in each group, with quantitative analysis of skeletal muscle wet weight. Groups: Vehicle shCtrl (empty control virus with vehicle intragastric gavage group), Dex shCtrl (empty control virus with dexamethasone intragastric gavage group), Dex shLcn2 (skeletal muscle-specific LCN2 knockdown with dexamethasone intragastric gavage group) (n=6 per group, Scale bar, 1 cm). (B) Quantitative analysis of skeletal muscle function in mice from each group, including grid-hanging endurance test, grip strength test, and exhaustive running distance on a treadmill (n=6 per group). (C) H&E staining of gastrocnemius muscle sections from mice in each group, with the frequency distribution of myofiber CSA (Scale bar, 100 μm). (D) Quantitative real-time PCR analysis of relative *Lcn2* mRNA levels in gastrocnemius muscles from the Vehicle shCtrl, Dex shCtrl and Dex shLcn2 groups, with β-actin as the reference gene (n=4 per group). (E) Quantitative real-time PCR validation of the relative mRNA levels of core ECM structural genes (Col1a1, Col3a1, Col4a1, Col6a1, Integrin α11) in gastrocnemius muscles from each group, with β-actin as the reference gene (n=4 per group). **(F)** Western blot analysis of LCN2, Collagen I, total and phosphorylated core proteins of the Akt-mTOR pathway, muscle-specific atrophy ubiquitin ligases Atrogin-1 and MuRF1, and global protein ubiquitination levels in gastrocnemius muscles from each group, with corresponding quantitative analysis of protein levels. GAPDH was used as the loading control (n=3 per group). All quantitative data are presented as mean ± standard error of the mean (SEM). **p*<0.05, ***p*<0.01, ****p*<0.001.

**Table S1 Western blot and ELISA materials**

| Reagent | Source | Catalog Number |
| --- | --- | --- |
| **Western blot materials** | | |
| Lipocalin-2/NGAL Antibody | R＆D Systems | AF1857 |
| FBXO32/Atrogin-1 Antibody | Proteintech | 67172-1-Ig |
| TRIM63/MuRF1 Antibody | Proteintech | 55456-1-AP |
| Collagen I Antibody | Immunoway | YM8576 |
| Phospho-Akt (Ser473) Antibody | Cell Signaling Technology | 4060S |
| Akt Antibody | Cell Signaling Technology | 9272S |
| MYH3 Antibody | Proteintech | 22287-1-AP |
| Ubiquitin (P4D1) Antibody | Cell Signaling Technology | 3936S |
| Phospho-mTOR (Ser2448) Antibody | Proteintech | 67778-1-Ig |
| mTOR Antibody | Proteintech | 66888-1-Ig |
| Phospho-p70 S6 Kinase (Thr389) Antibody | Cell Signaling Technology | 9234S |
| p70 S6 Kinase Antibody | Cell Signaling Technology | 9202S |
| Phospho-4E-BP1 (Thr37/46) Antibody | Cell Signaling Technology | 2855S |
| 4E-BP1 Antibody | Cell Signaling Technology | 9452S |
| Anti-Puromycin Antibody | Sigma-Aldrich | MABE343 |
| Phospho-PI3K Antibody | Cell Signaling Technology | 13857S |
| PI3K Antibody | Proteintech | 67071-1-Ig |
| MMP8 Antibody | Abcam | [AB53017](https://www.abcam.cn/products/primary-antibodies/mmp8-antibody-ab53017) |
| MMP9 Antibody | Abcam | AB76003 |
| Phospho-FAK (Tyr397) Antibody | Immunoway | YP0739 |
| FAK Antibody | zenbio | #R24276 |
| Phospho-FoXO3 (Ser253) Antibody | Cell Signaling Technology | 9466S |
| FoXO3 Antibody | Cell Signaling Technology | 2497S |
| Anti-GAPDH Antibody | Sino Biological Inc | 100242-MM05 |
| HRP conjugated goat anti-mouse IgG (H+L) | Proteintech | SA00001-1 |
| HRP conjugated goat anti-rabbit IgG (H+L) | Proteintech | SA00001-2 |
| PMSF | Servicebio | G2008 |
| Phosphatase Inhibitor | AmBeed | A2679231 |
| RIPA buffer | Beyotime Biotechnology | P0013B |
| SDS-PAGE protein loading buffer | BestEnzymes Biotech | EG21323S |
| Thermo Scientific™ PageRuler | ThermoFisher Scientific | 26616 |
| PVDF membranes | Millipore | ISEQ00010 |
| ECL reagent | ABBKINE | BMU102-CN |
| **ELISA materials** | | |
| Mouse lipocalin-2/NGAL ELISA kit | Elabscience | E-EL-M0828 |
| Human lipocalin-2/NGAL ELISA kit | Elabscience | E-EL-H6127 |

**Table S2**. List of specific primers used for real-time quantitative PCR gene expression analysis and ChIP analysis.

| Gene symbol | Forward primer 5’-3’ | Reverse primer 5’-3’ | Species |
| --- | --- | --- | --- |
| LCN2 | GCAGGTGGTACGTTGTGGG | CTCTTGTAGCTCATAGATGGTGC | mouse |
| COL1a1 | CTGGCGGTTCAGGTCCAAT | TTCCAGGCAATCCACGAGC | mouse |
| COL3a1 | CCTGGCTCAAATGGCTCAC | CAGGACTGCCGTTATTCCCG | mouse |
| COL4a1 | CTGGCACAAAAGGGACGAG | ACGTGGCCGAGAATTTCACC | mouse |
| COL6a1 | CCGACTGCGCCATTAAGAAG | CCCCGCATGGTTCCTTGTAG | mouse |
| Integrin α6 | TGCAGAGGGCGAACAGAAC | GCACACGTCACCACTTTGC | mouse |
| Integrin α11 | GAAGATGCCGTCCATGAGTTC | CTCCGAGCGTGCAAATTCAAT | mouse |
| Integrin β3 | ACCCGGAGCTAGGACTGAGA | CAGGTGAGCTGCATCCTTCT | mouse |
| Atrogin-1 | AGCGCTTCTTGGATGAGAAA | GGCAGTCGAGAAGTCCAGTC | mouse |
| MuRF1 | TGCCTGGAGATGTTTACCAAGC | AAACGACCTCCAGACATGGACA | mouse |
| FoxO1 | GCACCTTTGGAGAATGTGGT | CTGAGCCTGGGTCATTTTGT | mouse |
| FoxO3 | AAAGTTGCCCCGAAGGTTAAA | GAGCATAGTTTTCCAGAGAAGCA | mouse |
| MMP8 | TGGTGATTTCTTGCTAACCCC | TACACTCCAGACGTGAAAAGC | mouse |
| MMP9 | CTGGACAGCCAGACACTAAAG | CTCGCGGCAAGTCTTCAGAG | mouse |
| Pik3r1 | ACACCACGGTTTGGACTATGG | GGCTACAGTAGTGGGCTTGG | mouse |
| Ppp2r2d | GGAAGCCGACATCATCTCCAC | GTGAGCGCGGCCTTTATTCT | mouse |
| Ddit4 | CAAGGCAAGAGCTGCCATAG | CCGGTACTTAGCGTCAGGG | mouse |
| Eif4ebp1 | GGGGACTACAGCACCACTC | CTCATCGCTGGTAGGGCTA | mouse |
| LCN2 chip primer | GGTATTGGACACTTCCA | AGACCCTGTGCAGCTTC | mouse |
| LCN2 | CCACCTCAGACCTGATCCCA | CCCCTGGAATTGGTTGTCCTG | human |
| COL1a1 | GAGGGCCAAGACGAAGACATC | CAGATCACGTCATCGCACAAC | human |
| COL3a1 | GGAGCTGGCTACTTCTCGC | GGGAACATCCTCCTTCAACAG | human |
| COL4a1 | GGACTACCTGGAACAAAAGGG | GCCAAGTATCTCACCTGGATCA | human |
| COL6a1 | AGGGCTACAAGGAACCCTGT | CACCGAGAAGACTTTGACGC | human |
| Integrin α11 | GTGGCAATAAGTGGCTGGTC | GTTCCCGTGGATCACTGGAC | human |
